# Quantification of pulmonary involvement in COVID-19 pneumonia by means of a cascade of two U-nets: training and assessment on multiple datasets using different annotation criteria

**DOI:** 10.1007/s11548-021-02501-2

**Published:** 2021-10-26

**Authors:** Francesca Lizzi, Abramo Agosti, Francesca Brero, Raffaella Fiamma Cabini, Maria Evelina Fantacci, Silvia Figini, Alessandro Lascialfari, Francesco Laruina, Piernicola Oliva, Stefano Piffer, Ian Postuma, Lisa Rinaldi, Cinzia Talamonti, Alessandra Retico

**Affiliations:** 1 Scuola Normale Superiore, Pisa, Italy; 2 National Institute of Nuclear Physics (INFN), Pisa division, Pisa, Italy; 3 Department of Physics, University of Pisa, Pisa, Italy; 4 INFN, Pavia division, Pavia, Italy; 5 Department of Physics, University of Pavia, Pavia, Italy; 6 Department of Mathematics, University of Pavia, Pavia, Italy; 7 Department of Biomedical Experimental Clinical Science “M. Serio”, University of Florence, Florence, Italy; 8 Department of Chemistry and Pharmacy, University of Sassari, Sassari, Italy; 9 INFN, Cagliari division, Cagliari, Italy; 10 INFN, Florence division, Florence, Italy; 11 Department of Social and Political Science, University of Pavia, Pavia, Italy

**Keywords:** COVID-19, Chest Computed Tomography, Ground-glass opacities, Segmentation, Machine Learning, U-net

## Abstract

**Purpose:**

This study aims at exploiting artificial intelligence (AI) for the identification, segmentation and quantification of COVID-19 pulmonary lesions. The limited data availability and the annotation quality are relevant factors in training AI-methods. We investigated the effects of using multiple datasets, heterogeneously populated and annotated according to different criteria.

**Methods:**

We developed an automated analysis pipeline, the *LungQuant* system, based on a cascade of two U-nets. The first one (U-net$_1$) is devoted to the identification of the lung parenchyma; the second one (U-net$_2$) acts on a bounding box enclosing the segmented lungs to identify the areas affected by COVID-19 lesions. Different public datasets were used to train the U-nets and to evaluate their segmentation performances, which have been quantified in terms of the Dice Similarity Coefficients. The accuracy in predicting the CT-Severity Score (CT-SS) of the *LungQuant* system has been also evaluated.

**Results:**

Both the volumetric DSC (vDSC) and the accuracy showed a dependency on the annotation quality of the released data samples. On an independent dataset (COVID-19-CT-Seg), both the vDSC and the surface DSC (sDSC) were measured between the masks predicted by *LungQuant* system and the reference ones. The vDSC (sDSC) values of 0.95±0.01 and 0.66±0.13 (0.95±0.02 and 0.76±0.18, with 5 mm tolerance) were obtained for the segmentation of lungs and COVID-19 lesions, respectively. The system achieved an accuracy of 90% in CT-SS identification on this benchmark dataset.

**Conclusion:**

We analysed the impact of using data samples with different annotation criteria in training an AI-based quantification system for pulmonary involvement in COVID-19 pneumonia. In terms of vDSC measures, the U-net segmentation strongly depends on the quality of the lesion annotations. Nevertheless, the CT-SS can be accurately predicted on independent test sets, demonstrating the satisfactory generalization ability of the *LungQuant*.

**Supplementary Information:**

The online version supplementary material available at 10.1007/s11548-021-02501-2.

## Introduction

The task of segmenting the abnormalities of the lung parenchyma related to COVID-19 infection is a typical segmentation problem that can be addressed with methods based on Deep Learning (DL). CT findings of patients with COVID-19 infection may include bilateral distribution of ground-glass opacifications (GGO), consolidations, crazy-paving patterns, reversed halo sign and vascular enlargement [2]. Due to the extremely heterogeneous appearance of COVID-19 lesions in density, textural pattern, global shape and location in the lung, an analytical approach is definitely hard to code. The potential of DL-based segmentation approaches is particularly suited in this case, provided that a sufficient number of annotated examples are available for training the models. Few fully automated software tools devoted to this task have been recently proposed [4, 10, 11]. Lessmann et al. [10] developed a U-net model for lesion segmentation trained on semi-automatically annotated COVID-19 cases. The output of this system was then combined with the lung lobe segmentation algorithm reported in Xie et al. [14]. The approach proposed in Fang et al. [4] implements the automated lung segmentation method provided in the work of Hofmanninger et al. [7], together with a lesion segmentation strategy based on multiscale feature extraction [5]. The specific problem related to the development of fully automated DL-based segmentation strategies with limited annotated data samples has been explicitly tackled by Ma et al. [11]. The authors studied how to train and evaluate a DL-based system for lung and COVID-19 lesion segmentation on poorly populated samples of CT scans. They also made the data publicly available, allowing for a fair comparison with their system.

In this work, we present a DL-based fully automated system to segment both lungs and lesions associated with COVID-19 pneumonia, the *LungQuant* system, which provides the part of lung volume compromised by the infection. We extended the study proposed by Ma et al. [11] focusing our efforts in investigating and discussing the impact of using different datasets and different labelling styles. Data can be highly variable in terms of acquisition protocols and machines when they are gathered from different sources. This poses a serious problem of dependence of the segmentation performances on the training sample characteristics. Despite that advanced data harmonization strategies could mitigate this problem [6], this approach is not applicable in absence of data acquisition information, as it is in this study for the available CT data. Nevertheless, DL methods, when trained with sufficiently large samples of heterogeneous data, can acquire the desired generalization ability by themselves. In our analysis, we implemented an inter-sample cross-validation method to train, test and evaluate the generalization ability of the *LungQuant* DL-based segmentation pipeline across different available datasets. Finally, we also quantified the effect of using larger datasets to train, validate and test this kind of algorithm.

## Material and Methods

### Datasets

We used only publicly available datasets in order to make our results easily verifiable and reproducible. Five different datasets have been used to train and evaluate our segmentation pipeline. Most of them include image annotations, but each annotation has been associated with patients using different criteria. In Table 1, a summary of available labels for each dataset is reported.

**Table 1:** A summary of the datasets used in this study. The CT Severity Score (CT-SS) information is not available for all datasets, but it can be computed for data which has both lung masks and ground-glass opacification (GGO) masks

Dataset name	Lung	GGO	CT-SS	N. of
	mask	mask		cases
Plethora [8]	Yes	No	No	402
Lung CT Segmentation Challenge [15]	Yes	No	No	60
COVID-19 Challenge [1]	No	Yes	No	199
MosMed [12]	No	No	No	1110
MosMed (annotated subsample)	No	Yes	Inferable	50
MosMed (in-house annotated subsample)	Yes	No	No	91
COVID-19-CT-Seg [11]	Yes	Yes	Inferable	10

The lung segmentation problem has been tackled using a wide representation of the population and three different datasets: the Plethora, the Lung CT Segmentation Challenge and a subset of the MosMed dataset. On the other hand, the number of samples that are publicly available for COVID-19 infection segmentation may not be sufficient to obtain good performances on this task. The currently available data, provided along with infection annotations, have been labelled following different guidelines and released in NifTI format. They do not contain complete acquisition and population information, and they have been stored according to different criteria (see the Supplementary Materials for further details). Some of the choices made during the DICOM to NifTI conversion may strongly affect the quality of data. For example, the MosMed dataset as described by Morozov et al. [12] preserves only one slice out of ten during this conversion. This operation results in a significantly loss of resolution with respect to the COVID-19 Challenge dataset. Questioning how much such conversion influences the quantitative analysis is important to improve not only the performance but also the possibility of comparing DL algorithm in a fair modality.

### *LungQuant*: a DL based quantification analysis pipeline

The analysis pipeline, which is hereafter referred to as the *LungQuant* system, provides in output the lung and COVID-19 infection segmentation masks, the percentage P of lung volume affected by COVID-19 lesions and the corresponding CT-SS (CT-SS $=$ 1 for P< 5%, CT-SS $=$ 2 for 5% $\le$ P< 25%, CT-SS $=$ 3 for 25% $\le$ P< 50%, CT-SS $=$ 4 for 50% $\le$ P< 75%, CT-SS $=$ 5 for P $\ge$ 75%).

**Fig. 1:**
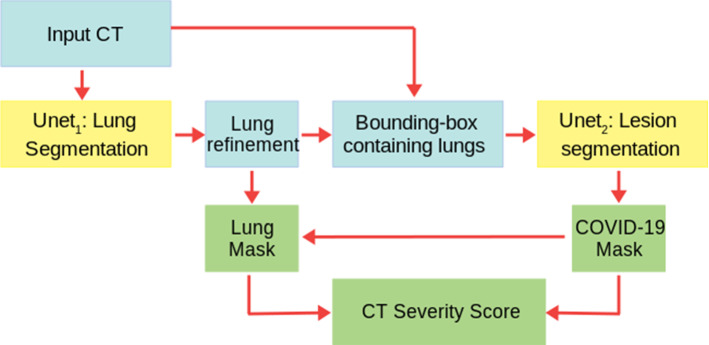
A summary of the whole analysis pipeline: the input CT scans are used to train U-net$_1$, which is devoted to lung segmentation; its output is refined by a morphology-based method. A bounding box containing the segmented lungs is made and applied to all CT scans for training U-net$_2$, which is devoted to COVID-19 lesion segmentation. Finally, the output of U-net$_2$ is the definitive COVID-19 lesion mask, whereas the definitive lung mask is obtained as the union between the outputs of U-net$_1$ and U-net$_2$. The ratio between the COVID-19 lesion mask and the lung mask provides the CT-SS for each patient

A summary of our image analysis pipeline is reported in Fig. 1. The central analysis module is a U-net for image segmentation [13] (see Sec. U-net), which is implemented in a cascade of two different U-nets: the first network, U-net$_1$, is trained to segment the lung and the second one, U-net$_2$, is trained to segment the COVID lesions in the CT scans.

#### U-net

**Fig. 2:**
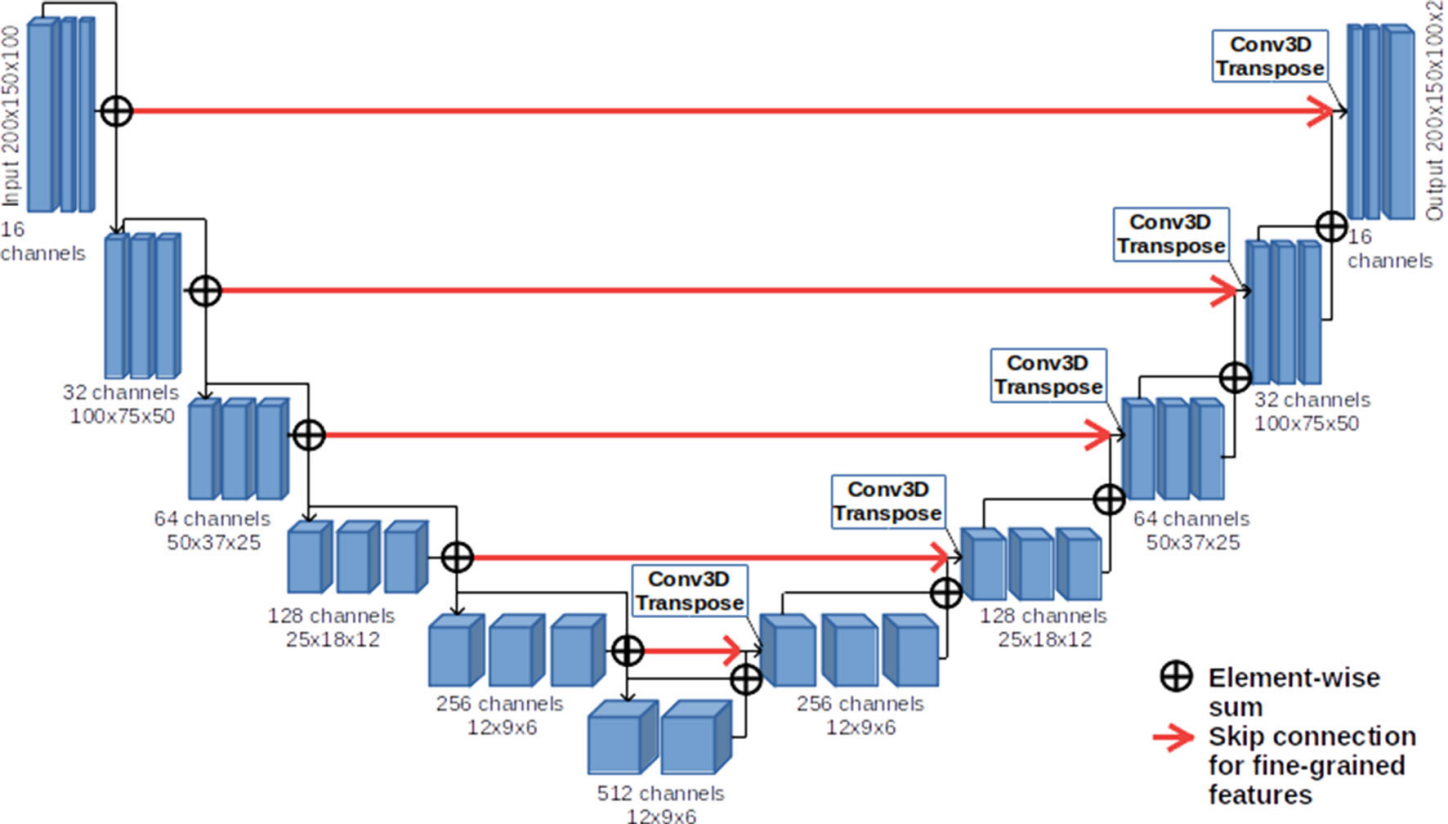
U-net scheme: the neural network is made of 6 levels of depth. In the compression path (left), the input is processed through convolutions, activation layers (ReLu) and instance normalization layers, while in the decompression one (right), in addition to those already mentioned, 3D Transpose Convolution (de-convolution) layers are also introduced

For both lung and COVID-19 lesion segmentation, we implemented a U-net using Keras [3], a Python DL API that uses Tensorflow as backend. In Fig. 2, a simplified scheme of our U-net is reported.

Each block of layers in the compression path (left) is made by 3 convolutional layers, ReLu activation functions and instance normalization layers. The input of each block is added to the block output in order to implement a residual connection. In the decompression path (right), one convolutional layer has been replaced by a de-convolutional layer to upsample the images to the input size. In the last layer of the U-nets, a softmax is applied to the final feature map, and then, the loss is computed.

#### The U-net cascade for lesion quantification and severity score assignment

The input CT scans, whose number of slices is highly variable, have been resampled to matrices of $200 \times 150 \times 100$ voxels and then used to train U-net$_1$, which is devoted to lung segmentation, using the three datasets containing original CT scans and lung masks (see Table 1). The output of U-net$_1$ was refined using a connected component labelling strategy to remove small regions of the segmented mask not connected with the main objects identified as the lungs. We identified the connected components in the lung masks generated by U-net$_1$, and we excluded those components whose number of voxels was below an empirically fixed threshold (see Supplementary Materials for further details). We then built for each CT a bounding box enclosing the refined segmented lungs, adding a conservative padding of 2.5 cm. The bounding boxes were used to crop the training images for U-net$_2$, which has the same architecture as U-net$_1$. Training U-net$_2$ to recognize the COVID-19 lesions on a conservative bounding box has two main advantages: it allows to restrict the action volume of the U-net to the region where the lung parenchyma is supposed to be, thus avoiding false-positive findings outside the chest; it facilitates the U-net training phase, as the dimensions of the lungs of different patients are standardized to focus the U-net learning process on the textural patterns characterizing the COVID-19 lesions. The cropped images were resized to a matrix of $200 \times 150 \times 100$ voxels. We applied a windowing on the grey-level values of the CT scans to optimize the image contrast for the two segmentation problems: the [− 1000, 1000] HU window range for the U-net$_1$ and the [− 1000, 300] HU range for U-net$_2$. The first window highlights the contrast between the lung parenchyma and the surrounding tissues, whereas the second one enhances the heterogeneous structure of the lung abnormalities related to the COVID-19 infection. We implemented a data augmentation strategy, relying on the most commonly used data augmentation techniques for DL (see Supplementary Materials for further details) to overcome the problem of having a limited amount of labelled data. We transformed the images with rotations, zooming, elastic transformations and adding Gaussian noise.

The *LungQuant* system returns the infection mask as the output of U-net$_2$ and the lung mask as the union between the output of U-net$_1$ and U-net$_2$. This choice has been made *a priori* by design, as U-net$_1$ has been trained to segment the lungs relying on the available annotated data, which are almost totally of patients not affected by COVID-19 pneumonia. Thus, U-net$_1$ is expected to be unable to accurately segment the areas affected by GGO or consolidations; as also these areas are part of the lungs, they should be instead included in the mask.

Lastly, once lung and lesion masks have been identified, the *LungQuant* system computes the percentage of lung volume affected by COVID-19 lesions as the ratio between the volume of the infection mask and the volume of the lung mask and converts it into the corresponding CT severity score.

### Training details and evaluation strategy for the U-nets

Both U-net${_1}$ and U-net${_2}$ have been evaluated using the volumetric Dice Similarity Coefficients (vDSC). U-net${_1}$ has been trained with the vDSC as loss function, while U-net${_2}$ has been trained using the sum of the vDSC and a weighted cross-entropy as error function in order to balance the number of voxels representing lesions and the background (see Supplementary Materials for further details). The performances of the whole system have been evaluated also with the surface Dice Similarity Coefficient (sDSC) for different values of tolerance [9].

#### Cross-validation strategy

To train, validate and test the performances of the two U-nets, we partitioned the datasets into training, validation and test sets. We then evaluated the network performance separately and globally. U-net$_2$ has been trained twice, i.e. on the 60% and 90% of the CT scans of COVID-19-Challenge and Mosmed datasets to investigate the effect of maximizing the training set size on the lesion segmentation. The amount of CT scan used for train, validation and test sets for each U-net is reported in Table 2. To evaluate the ability of the trained networks to predict the percentage of the affected lung parenchyma and thus the CT-SS classification, we used a completely independent set consisting of 10 CT scans from the COVID-19-CT-Seg dataset, which is the only publicly available dataset containing both lung and infection mask annotations.

**Table 2:** Number of CT scans assigned to the train, validation (val) and test sets used during the training and performance assessment of the U-net$_1$ and the U-net$_2$ networks

**U-net**$_1$	Train	Val	Test
Plethora	319	40	40
MosMed (91 CT-0)	55	18	18
LCTSC	36	12	12
COVID-19-CT-Seg	–	–	10

**U-net**$_2^{60\%}$	Train (60%)	Val (20%)	Test
COVID-19 Challenge	119	40	40
MosMed (50 CT-1)	30	10	10
COVID-19-CT-Seg	–	–	10

**U-net**$_2^{90\%}$	Train (90%)	Val (10%)	Test
COVID-19 Challenge	179	20	–
MosMed (50 CT-1)	45	5	–
COVID-19-CT-Seg	–	–	10

## Results

In this section, we report, first, the performance achieved by U-net$_1$ and U-net$_2$, then, the quantification performance of the integrated *LungQuant* system, evaluated on a completely independent test set. We trained both the U-nets for 300 epochs on a NVIDIA V100 GPU using ADAM as optimizer and we kept the models trained at the epoch where the best evaluation metric on the validation set was obtained.

### U-net$_1$: Lung segmentation performance

U-net$_1$ for lung segmentation was trained and validated using three different datasets, as specified in Table 2. Then, we tested U-net$_1$ on each of the three independent test sets and we reported in Table 3 the performance achieved in terms of vDSC, computed between the segmented masks and the reference ones, both separately for each dataset and globally.

The evaluation of the lung segmentation performances was made in three cases: (1) on CT scans and masks resized to the $200 \times 150 \times 100$ voxel array size; (2) on CT scans and masks in the original size before undergoing the morphological refinement; (3) on CT scans and masks in the original size and after the morphological refinement. Even if segmentation refinement has a small effect on vDSC, since it is a volume-based metric, as shown in Table 3, it is a fundamental step to allow the definition of precise bounding boxes enclosing the lungs and thus to facilitate the U-net$_2$ learning process.

**Table 3:** Performances achieved by U-net$_1$ in lung segmentation on different test sets, evaluated in terms of the vDSC at three successive stages of the segmentation procedure

Test set	Masks of U-net size	Masks before refinement	Masks after refinement
	vDSC	vDSC	vDSC
Plethora	0.96 ± 0.02	0.95 ± 0.02	0.95 ± 0.04
MosMed	0.97 ± 0.02	0.97 ± 0.02	0.97 ± 0.02
LCTSC	0.96 ± 0.03	0.95 ± 0.03	0.96 ± 0.01
COVID-19-CT-Seg	0.96 ± 0.01	0.95 ± 0.01	0.95 ± 0.01

### U-net$_2$: COVID-19 lesion segmentation performance

U-net$_2$ for COVID-19 lesion segmentation has been trained and evaluated separately on the COVID-19-Challenge dataset and on the annotated subset of the MosMed dataset, following the train/validation/test partitioning reported in Table 2. The segmentation performances achieved on the test sets are reported in terms of the vDSC in Table 4, according to the cross-sample validation scheme.

**Table 4:** Performances achieved by U-net$_2$ in COVID-19 lesion segmentation, evaluated in terms of the vDSC

U-net	Trained on	Test set	U-net size	Original CT size
			(vDSC)	(vDSC)
U-net$_2^{60\%}$	COVID-19 challenge	COVID-19 challenge	0.51 ± 0.24	0.51 ± 0.25
	COVID-19 Challenge	MosMed	0.39 ± 0.19	0.40 ± 0.19
	MosMed	MosMed	0.54 ± 0.22	0.55 ± 0.22
	MosMed	COVID-19 challenge	0.25 ± 0.23	0.25 ± 0.23
	COVID-19 challenge	COVID-19 challenge	0.49 ± 0.21	0.50 ± 0.21
	+ MosMed	+ MosMed		
U-net$_2^{90\%}$	COVID-19 challenge	COVID-19 challenge	0.64 ± 0.23	0.65 ± 0.23
	+ MosMed	+ MosMed		

The composition of the train and test sets is reported in Table 2

As expected, the U-net$_2$ performances are higher when both the training set and independent test sets belong to the same data cohort. By contrast, when a U-net$_2$ is trained on COVID-19-Challenge data and tested on Mosmed (and the other way around), performances significantly decrease. This effect is related to different criteria used to both collect and annotate the data. We obtained a better result with the U-net$_{2}$ trained on the COVID-19 Challenge dataset and tested on the MosMed test set, since the network has been trained on a larger data sample and hence it has a higher generalization capability. The best segmentation performances have been obtained by the U-net$_2$ trained using the 90$\%$ of the available data, U-net$_2^{90\%}$, which reaches a vDSC of 0.65 ± 0.23 on the test set. This result suggests the need to train U-net models on the largest possible data samples in order to achieve higher segmentation performance.

**Table 5:** Performances of the *LungQuant* system on the independent COVID-19-CT-Seg test dataset. The vDSC and sDSC computed between the lung and lesion reference masks and those predicted by the *LunQuant* system are reported

Metrics	Lung segmentation			
	vDSC	sDSC (1 mm)	sDSC (5 mm)	sDSC (10 mm)
*LungQuant* (U-net$_2^{60\%})$	0.96 ± 0.01	0.66 ± 0.09	0.95 ± 0.02	0.98 ± 0.01
*LungQuant* (U-net$_2^{90\%})$	0.95 ± 0.01	0.65 ± 0.09	0.95 ± 0.02	0.98 ± 0.01
Infection Segmentation				
*LungQuant* (U-net$_2^{60\%})$	0.62 ± 0.09	0.29 ± 0.06	0.75 ± 0.11	0.90 ± 0.09
*LungQuant* (U-net$_2^{90\%})$	0.66 ± 0.13	0.36 ± 0.13	0.76 ± 0.18	0.87 ± 0.13

**Fig. 3:**
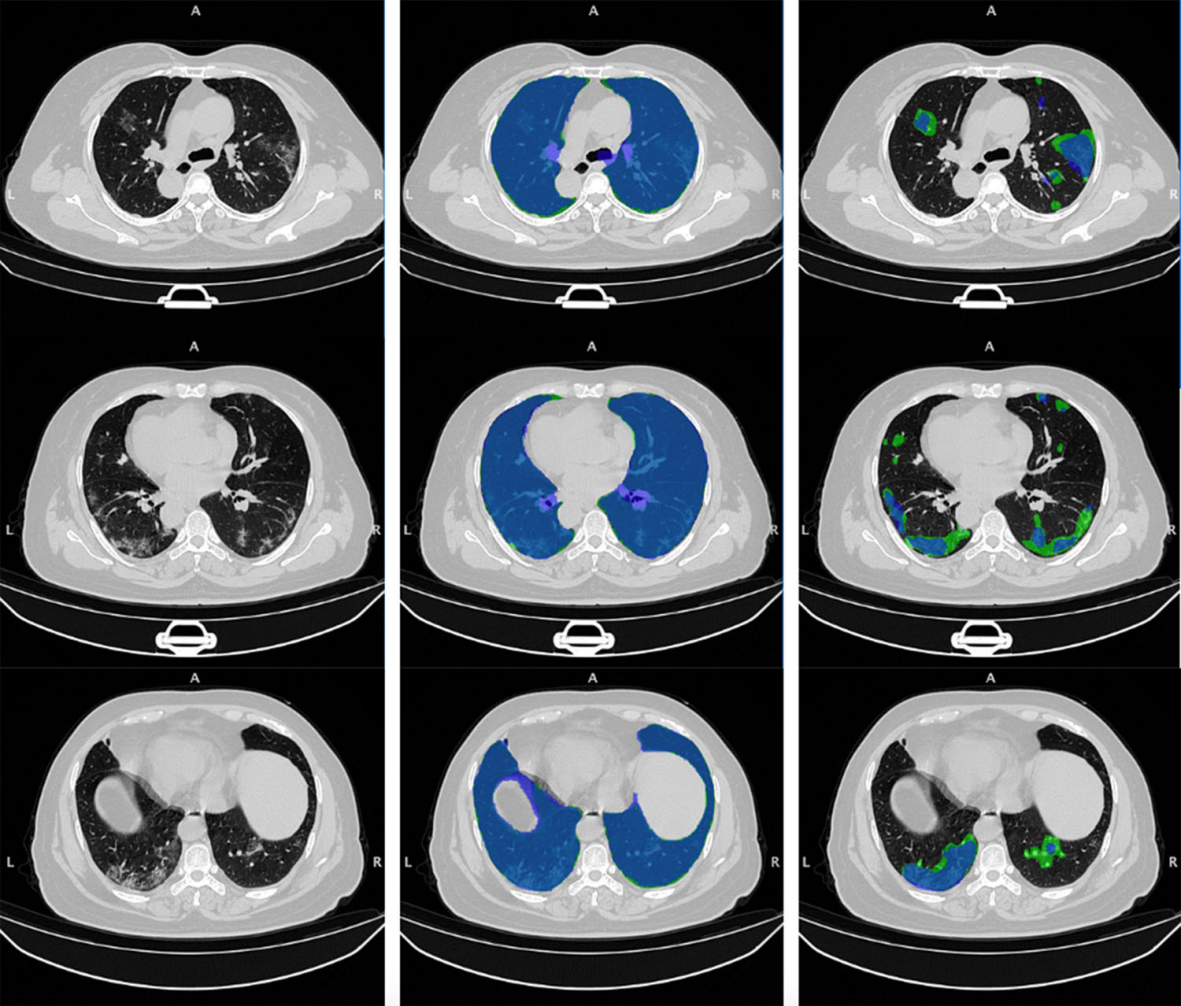
On the rows: three axial slices of the first CT scan on the COVID-19-CT-Seg test dataset (*coronacases*001.*nii*) are shown. On the columns: original images (left); overlays between the predicted and the reference lung (centre) and COVID-19 lesion (right) masks. The reference masks are in green, while the predicted ones, obtained by the *LungQuant* system integrating U-net$_2^{90\%}$,are in blue

### Evaluation of the quantification performance of the *LungQuant* system on a completely independent set

#### Evaluation of lung and COVID-19 lesion segmentations

Once the two U-nets have been trained and the whole analysis pipeline has been integrated into the *LungQuant* system, we tested it on a completely independent set (COVID-19-CT-Seg dataset) of CT scans. The performances of the whole process were quantified both in terms of vDSC and sDSC with tolerance values of 1, 5 and 10 mm (Table 5). A very good overlap between the predicted and reference lung masks is observable in terms of vDSC, whereas the sDSC values are highly dependent on tolerance values, ranging from moderate to very good agreement measures. Regarding the lesion masks, a moderate overlap is measured between the predicted and reference lesion masks in terms of vDSC, whereas the sDSC returns measures extremely dependent on tolerance values that span from limited to moderately good and ultimately satisfactory performances for tolerance values of 1 mm, 5 mm and 10 mm, respectively.. Figure 3 allows for a visual comparison between the lung and lesion masks provided by the *LungQuant* system integrating U-net$_2^{90\%}$ and the reference ones.

#### Percentage of affected lung volume and CT-SS estimation

**Fig. 4:**
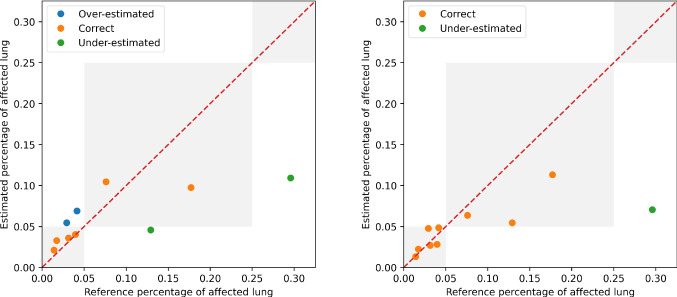
Estimated percentages P of affected lung volume versus the ground truth percentages, as obtained by the *LungQuant* system integrating U-net$_2^{60\%}$ (left) and U-net$_2^{90\%}$ (right). The grey areas in the plot backgrounds guide the eye to recognize the CT-SS values assigned to each value of P (from left to right: CT-SS $=$ 1, CT-SS $=$ 2, CT-SS $=$ 3)

The lung and lesion masks provided by the *LungQuant* system can be further processed to derive the physical volumes of each mask and the ratios between the lesion and lung volumes. We show in Fig. 4 the relationship between the percentage of lung involvement as predicted by the *LungQuant* system vs. the corresponding values for the reference masks of the fully independent test set COVID-19-CT-Seg, for both the *LungQuant* systems with the U-net$_2^{60\%}$ and the U-net$_2^{90\%}$. Despite the limited range of samples to carry out this test, an agreement between the *LungQuant* system output and the reference values is observed for both U-net$_2^{60\%}$ and U-net$_2^{90\%}$. In terms of the mean absolute error (MAE) among the estimated and the reference percentages of affected lung volume (P), we obtained a Mean Absolute Error equal to MAE $=$ 4.6% for the LungQuant system with U-net$_2^{60\%}$ and MAE $=$ 4.2% for the system with U-net$_2^{90\%}$.

The accuracy in assigning the correct CT-SS class is reported in Table 6, together with the number of misclassified cases, for the 10 cases of the COVID-19-CT-Seg dataset. The best accuracy achieved by *LungQuant* is of 90% with U-net$_2^{90\%}$. In all cases, the system misclassifies the examples by 1 class at most.

**Table 6:** Classification performances of the whole system in predicting CT Severity Score on MosMed and COVID-19-CT-Seg datasets. The number of misclassified cases is reported

U-net	Dataset	Accuracy	Misclassified	Misclassified
			by 1 class	by 2 classes
U-net$_2^{60\%}$	COVID-19-CT-Seg	6/10	4/10	0
U-net$_2^{90\%}$	COVID-19-CT-Seg	9/10	1/10	0

## Discussion and Conclusion

We developed a fully automated quantification pipeline, the *LungQuant* system, for the identification and segmentation of lungs and pulmonary lesions related to COVID-19 pneumonia in CT scans. The system returns the COVID-19 related lesions, the lung mask and the ratio between their volumes, which is converted into a CT Severity Score. The performance obtained against a voxel-wise segmentation ground truth was evaluated in terms of the vDSC, which provides a measure of the overlap between the predicted and the reference masks. The *LungQuant* system achieved a vDSC of 0.95 ± 0.01 in the lung segmentation task and of 0.66 ± 0.13 in segmenting the COVID-19 related lesions on the fully annotated publicly available benchmark COVID-19-CT-Seg dataset of 10 CT scans. The *LungQuant* has been evaluated also in terms of sDSC for different values of tolerance. The results obtained at a tolerance of 5 mm equal to $0.76\pm 0.18$ are satisfactory for our purpose, given the heterogeneity of the labelling process. Regarding the correct assignment of the CT-SS, the *LungQuant* system showed an accuracy of 90% on the completely independent test set COVID-19-CT-Seg. Despite that this result is encouraging, it was obtained on a rather small independent test set; thus, a broader validation on larger data sample with more heterogeneous composition in terms of disease severity is required. Training DL algorithms requires a huge amount of labelled data. The lung segmentation task has been made feasible in this work thanks to the use of lung CT datasets collected for purposes different from the study of COVID-19 pneumonia. Training a segmentation system on these samples had the effect that when we use the trained network to process the CT scan of a patient with COVID-19 lesions, especially in case ground glass opacities and consolidation are very severe, the lung segmentation is not accurate anymore. In order to overcome this problem, the proposed *LungQuant* system returns a lung mask which is the logical union between the output mask of the U-net$_{1}$ and the infection mask generated by the U-net$_{2}$. The *LungQuant* system can actually be improved whether lung masks annotation are available on subjects with COVID-19 lesions. A similar problem occurs for the segmentation of ground glass opacities and consolidations. The available data, in fact, are very unbalanced with respect to the severity of COVID-19 disease, and hence, the accuracy in segmenting the most severe case is worse. The current lack of a large dataset, collected by paying attention to adequately represent all categories of disease severity, limits the possibility to carry out accurate training of AI-based models. Finally, we found that the difference in the annotation and collection guidelines among datasets is an issue. Processing aggregated data from different sources can be difficult if labelling has been performed using different guidelines. CT scans should contain the acquisition parameters, usually stored in the DICOM header, when they are published. The lack of this information is a drawback of our study. If we had that data, we could study more in detail the dependence of the *LungQuant* performances on specific acquisition protocols or scanners. On the contrary, even with this information, it would not be possible to standardize the different annotation styles. The results of *LungQuant* (last 2 rows of Table 4) demonstrate its robustness across different datasets even without a dedicated preprocessing step to account for this information.

## Supplementary Information

Below is the link to the electronic supplementary material.

Supplementary material 1 (pdf 9932 KB)
