# Supplementary material for: Quantification of pulmonary involvement in COVID-19 pneumonia by means of a cascade of two U-nets: training and assessment on multiple datasets using different annotation criteria
Source: Int J Comput Assist Radiol Surg. 2021 Oct 26;17(2):229–37. doi: 10.1007/s11548-021-02501-2 (PMC8547130; doi:10.1007/s11548-021-02501-2)
Supplement: Supplementary file 1 — Supplementary material 1 (pdf 9932 KB) [file 11548_2021_2501_MOESM1_ESM.pdf]

Noname manuscript No.
(will be inserted by the editor)

SUPPLEMENTARY MATERIALS

Quantification of pulmonary involvement in COVID-19 pneumonia by means of a cascade of two U-nets: training and assessment on multiple datasets using different annotation criteria

Francesca Lizzi [1,2] et al.

August 28, 2021

1 Additional descriptions of Materials and Methods

1.1 Characteristics of the public datasets used in the study

1.1.1 The Plethora dataset

The PleThora dataset [4] is a chest CT scan collection with thoracic volume and pleural effusion segmentations, delineated on 402 CT studies of the Non-Small Cell Lung Cancer (NSCLC) radiomics dataset, available through the The Cancer Imaging Archive (TCIA) repository [3]. This dataset has been made publicly available to facilitate improvement of the automatic segmentation of lung cavities, which is typically the initial step in the development of automated or semi-automated algorithms for chest CT analysis. In fact, automatic lung identification struggles to perform consistently in subjects with lung diseases. The PleThora lung annotations have been produced with a U-net based algorithm trained on chest CT of subjects without cancer, manually corrected by a medical student and revised by a radiation oncologist or a radiologist.

1.1.2 The 2017 Lung CT Segmentation Challenge dataset

The Lung CT Segmentation Challenge (LCTSC) dataset consists of CT scans of 60 patients, acquired from 3 different institutions and made publicly available in the context of the 2017 Lung CT Segmentation Challenge [10]. Since the aim of this challenge was to foster the development of auto-segmentation methods for organs at risk in radiotherapy, the lung annotations followed the RTOG 1106 contouring atlas.

[1] Scuola Normale Superiore, Pisa;
[2] National Institute of Nuclear Physics (INFN), Pisa division, Pisa, IT

1.1.3 The 2020 COVID-19 Lung CT Lesion Segmentation Challenge dataset

The 2020 COVID-19 Lung CT Lesion Segmentation Challenge dataset (COVID-19 Challenge) is a public dataset consisting of unenhanced chest CT scans of 199 patients with positive RT-PCR for SARS-CoV-2 [1]. Each CT is accompanied with the ground truth annotations for COVID-19 lesions. Data has been provided in NIfTI format by The Multi-national NIH Consortium for CT AI in COVID-19 via the NCI TCIA public website [3]. Annotations have been made using a COVID-19 lesion segmentation model provided by NVIDIA, which takes a full CT chest volume and produces pixel-wise segmentations of COVID-19 lesions. These segmentations have been adjusted manually by a certified radiologists board, in order to give 3D consistency to lesion masks. The dataset annotation was made possible through the joint work of Children's National Hospital, NVIDIA and National Institutes of Health for the COVID-19-20 Lung CT Lesion Segmentation Grand Challenge.

The dataset and the annotations have been made available in the context of a MICCAI-endorsed international challenge (https://covid-segmentation.grand-challenge.org/) which had the aim to compare AI-based approaches to automated segmentation of COVID-19 lung lesions.

1.1.4 The MosMed dataset

MosMed [7] is a COVID-19 chest CT dataset collected by the Research and Practical Clinical Center for Diagnostics and Telemedicine Technologies of the Moscow Health Care Department. It includes CT studies taken from 1110 patients. Each study is represented by one series of images reconstructed into soft tissue mediastinal window. MosMed provides 5 labeled categories, based on the percentage of lung parenchyma affected by COVID-19 lesions. The 5 categories of lung involvement and their correspondence to the CT-SS scale are described in Table 1. The first category (CT-0) contains cases with normal lung tissue and no CT-signs of viral pneumonia, whereas the other categories contain GGO (CT-1 and CT-2) and both GGO and regions of consolidation in the higher classes (CT-3 and CT-4).

Table 1 MosMed severity categories defined on the basis of the percentage P of lung volume affected by COVID-19 lesions. The correspondence to the CT-SS scale is reported.

MosMed CT category	N. of cases	Percentage P of involved lung parenchyma	Corresponding CT-SS
0	254	$P = 0$	0
1	684	$0 < P \leq 25$	1, 2
2	125	$25 < P \leq 50$	3
3	45	$50 < P \leq 75$	4
4	2	$75 < P \leq 100$	5

A small subset of class CT-1 cases (50 patients) had been annotated by expert radiologists with the support of MedSeg software (2020 Artificial Intelligence AS). The annotations consist of binary masks in which white voxels represent both ground-glass opacifications and consolidations. Both CT scans and annotations were provided in NIfTI format. During the DICOM-to-NIfTI conversion process,

only one slice out of ten was preserved and, as a result, MosMed CT scans have a reduced total number of slices with respect to the other datasets.

1.1.5 The COVID-19-CT-Seg dataset

The COVID-19-CT-Seg dataset is a collection of CT scans taken from the Coronacases Initiative and Radiopaedia [6]. It contains 20 CT scans tested positive for COVID-19 infection. This public dataset contains both lung and infection annotations. The ground truth has been made in three steps: first, junior radiologists (1-5 years of experience) delineated lungs and infections annotations, then two radiologists (5-10 years of experience) refined the labels and finally the annotations have been verified and optimized by a senior radiologist (more than 10 years of experience in chest radiology). The annotations have been produced with the ITK-SNAP software. Ten CT images of this dataset were provided in 8-bit depth, therefore, we decided to not use them.

1.2 Additional training details and evaluation strategy for the U-nets

1.2.1 Evaluation metrics

The segmentation performances for both U-nets have been evaluated with the volumetric Dice Similarity Coefficient (vDSC), computed between the true mask volume (V_{true}) and the predicted mask volume ($V_{predict}$), and with the surface Dice Similarity Coefficient (sDSC), computed between the true surface (S_{true}), and the predicted one defined, ($S_{predict}$) [5], as follows;

$$\text{vDSC}_{metric} = \frac{2 \cdot |V_{true} \cap V_{predict}|}{|V_{true}| + |V_{pred}|} \tag{1}$$

$$\text{sDSC}_{metric} = \frac{2 \cdot |S_{true} \cap S_{predict}|}{|S_{true}| + |S_{pred}|} \tag{2}$$

The loss function used to train the U-net for lung segmentation is the vDSC loss, defined as follows

$$\text{vDSC}_{loss} = 1 - \frac{2 \cdot |M_{true} \cap M_{pred}|}{|M_{true}| + |M_{pred}|} \tag{3}$$

and computed only on the foreground (white voxels). We used this strategy in order to avoid giving excessive weight to the background (black voxels), since the number of black and white voxels is quite unbalanced in favor of the former.

For U-net$_2$, we used a loss function (L) consisting of the sum of the vDSC loss and a weighted cross-entropy (CE), defined as follows:

$$L = vDSC_{loss} + CE_{weighted} \tag{4}$$

$$CE_{weighted} = w(x) \sum_{x \in \Omega} log(M_{true}(x) \cdot M_{pred}(x)) \tag{5}$$

where $w(x)$ is the weight map which takes into account the frequency of white voxels, x is the current sample and Ω is the training set.

Since the background class is larger than the foreground class on the order 10^3, we computed the weight map $w(x)$ for each ground-truth segmentation to increase the relevance of the underrepresented class, following the approach described in [8]. The weight map was defined as $w(x) = w_0/f_j$ where f_j is the average number of voxels of the jth class over the entire training data set ($j = 0, 1$) and w_0 is the the average between the frequencies f_j.

1.2.2 Data augmentation

Data augmentation is a strategy to increase the size of the training set by synthetically generating additional training images through geometric transformations. This technique is particularly important to improve the generalization capability of the model, especially in the case of a limited number of training samples. In our work, we applied data augmentation during the data pre-processing phase (after defining the bounding boxes enclosing the segmented lungs) in order to generate a fixed number of augmented images for each original data. We chose an augmentation factor equal to 2 which means that the number of artificially generated images is twice the number of the original training set. For each image in the training set, two of the following geometric transformations were randomly chosen:

- Zooming. The CT image and the ground truth masks were zoomed in the axial plane, using a third-order spline interpolation and the k-nearest neighbor method, respectively. The zooming factor was randomly chosen among the following values: 1.05, 1.1, 1.15, 1.2.
- Rotation. The CT image and the ground truth mask were rotated in the axial plane, using a third-order spline interpolation and the k-nearest neighbor method, respectively. The rotation angle was randomly sampled among the following values: -15°, -10°, -5°, 5°, 10°, 15°.
- Gaussian noise. An array of noise terms randomly drawn from a normal distribution was added to the original CT image. For each image, the mean of the Gaussian distribution was randomly sampled in the [-400, 200] HU range and the standard deviation randomly chosen among 3 values: 25, 50, 75 HU.
- Elastic deformation. An elastic distortion was applied to the original 3D CT and mask arrays following the approach of Simard *et al.* [9]. This transformation has two parameters: the elasticity coefficient which we fixed to 12 and the scaling factor, fixed to 1000.
- Motion blurring. Slice by slice, we convolved the CT image with a linear kernel (i.e. ones along the central row and zero elsewhere for a matrix of size $k \times k$) through the function filter2D, defined in the OpenCV Python library [2], keeping the output image size the same as the input image. The filter is applied with a kernel size of 4, 3, and 3, in the anterior-posterior, latero-lateral and cranio-caudal direction, respectively.

An example of the application of these augmentation techniques to one CT scan of the dataset is provided in Fig. 1.

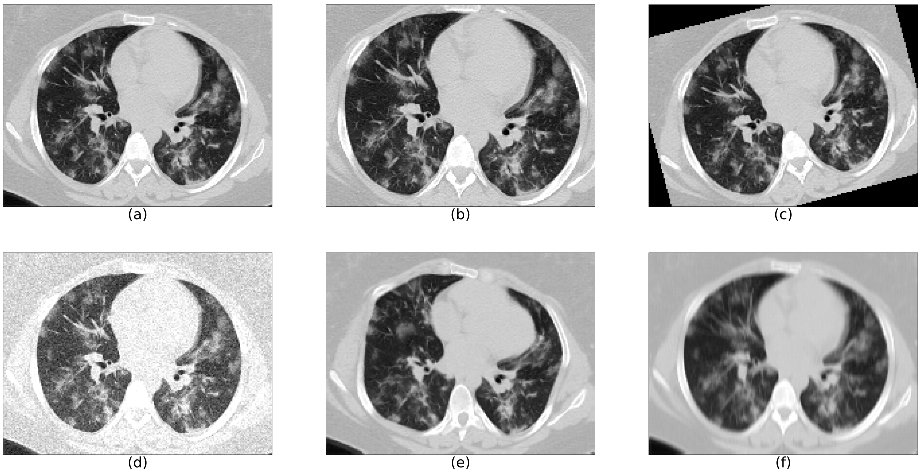

Fig. 1 Data augmentation to increase the diversity of dataset: a) Image without data augmentation; b) Zooming; c) Rotation; d) Gaussian noise; e) Elastic deformations; f) Motion blurring.

1.3 Morphological refinement of U-net$_1$ lung segmentation

In order to remove false-positive regions (*i.e.* voxels misclassified as lung parts), at first, we identified the connected components in the lung masks generated by U-net$_1$, then, we excluded those components whose number of voxels was below an empirically-fixed threshold. This threshold was set to the 40% of the foreground mask, and it was reduced to 30% whether the resulting number of voxels was found to be lower than the 65% of the initial mask provided by U-net$_1$. Figure 2 shows some examples of how this procedure works on real CT scans.

1.4 Generation of a set of reference lung segmentation for model training

As reported in Table 1 (main paper), the available datasets with lung mask annotations, which were necessary to train the U-net for lung segmentation, are mainly of subjects affected by lung cancer (Plethora and LCTSC datasets). To complement this sample with subjects without lesions, and, at the same time, to expose to U-net to the acquisition characteristics of the MosMed CT scans, we generated the lung mask annotations for a subset of subjects of the CT-0 MosMed category, i.e. that of subjects without COVID-19 lesions.

An in-house lung segmentation algorithm was developed for this purpose and implemented in *matlab* (The MathWorks, Inc.). It is based on the following steps: 1) CT windowing in the [-1000,1000] HU range; 2) rough segmentation of the lungs on a central coronal slice (Otsu binary thresholding and removal of components connected with the image border) to define the minimum and maximum axial coordinates of the lung region; 3) 2D rough segmentation of the lungs on each axial slice (same procedure as the previous step) to generate a 3D seed mask for the following step; 4) segmentation of the lung parenchyma by an active contour

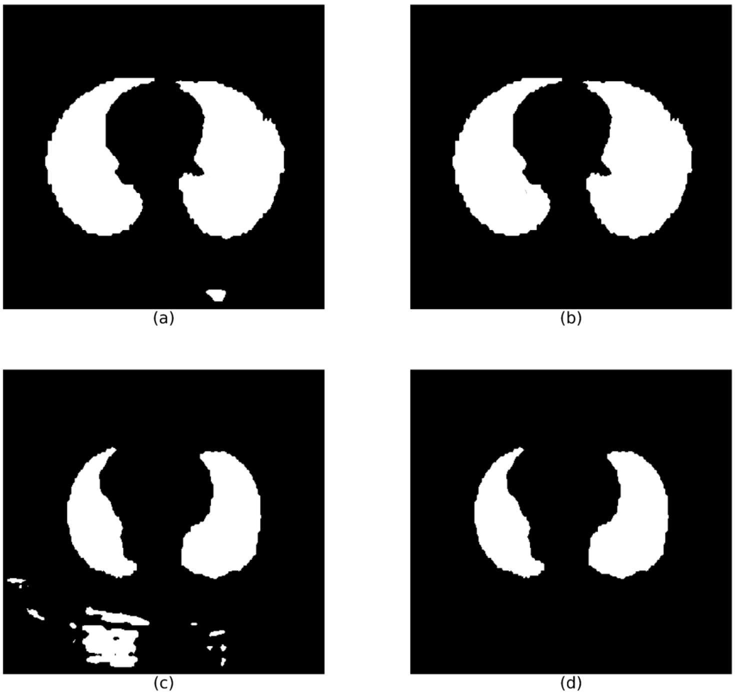

Fig. 2 Morphological refinement of the U-net$_1$ output: a) and c) lung masks as generated by U-net$_1$; b) and d) refined masks after the connected component selection.

model (*activecontour* matlab function); 5) filling holes (e.g. vessels and airway walls) with 3D morphological operators (*imclose* matlab function).

This algorithm, which accurately segments the lung parenchyma in absence of lesions, has very limited performance on CT scans of subjects with COVID-19 lesions.

References

1. An, P., Xu, S., Harmon, S.A., Turkbey, E.B., Sanford, T.H., Amalou, A., Kassin, M., Varble, N., Blain, M., Anderson, V., Patella, F., Carrafiello, G., Turkbey, B.T., Wood, B.J.: CT Images in COVID-19 (2020). DOI https://doi.org/10.7937/tcia.2020.gqry-nc81
2. Bradski, G.: The OpenCV Library. Dr. Dobb's Journal of Software Tools (2000)
3. Clark, K., Vendt, B., Smith, K., Freymann, J., Kirby, J., Koppel, P., Moore, S., Phillips, S., Maffitt, D., Pringle, M., Tarbox, L., Prior, F.: The cancer imaging archive (TCIA): Maintaining and operating a public information repository. Journal of Digital Imaging **26**(6), 1045–1057 (2013). DOI 10.1007/s10278-013-9622-7
4. Kiser, K.J., Ahmed, S., Stieb, S., Mohamed, A.S., Elhalawani, H., Park, P.Y., Doyle, N.S., Wang, B.J., Barman, A., Li, Z., Zheng, W.J., Fuller, C.D., Giancardo, L.: PleThora: Pleural effusion and thoracic cavity segmentations in diseased lungs for benchmarking chest CT processing pipelines. Medical Physics **47**(11), 5941–5952 (2020). DOI 10.1002/mp.14424
5. Kiser, K.J., Barman, A., Stieb, S., Fuller, C.D., Giancardo, L.: Novel Autosegmentation Spatial Similarity Metrics Capture the Time Required to Correct Segmentations Better Than Traditional Metrics in a Thoracic Cavity Segmentation Workflow. Journal of Digital

Imaging **34**(3), 541–553 (2021). DOI 10.1007/s10278-021-00460-3. URL https://doi.org/10.1007/s10278-021-00460-3

6. Ma, J., Wang, Y., An, X., Ge, C., Yu, Z., Chen, J., Zhu, Q., Dong, G., He, J., He, Z., Cao, T., Zhu, Y., Nie, Z., Yang, X.: Toward data-efficient learning: A benchmark for COVID-19 CT lung and infection segmentation. Medical Physics (2020). DOI 10.1002/mp.14676
7. Morozov, S.P., Andreychenko, A.E., Pavlov, N.A., Vladzymyrskyy, A.V., Ledikhova, N.V., Gombolevskiy, V.A., Blokhin, I.A., Gelezhe, P.B., Gonchar, A.V., Chernina, V.: MosMedData: Chest CT Scans with COVID-19 Related Findings Dataset. medRxiv p. 2020.05.20.20100362 (2020). DOI 10.1101/2020.05.20.20100362. URL http://medrxiv.org/content/early/2020/05/22/2020.05.20.20100362.abstract
8. Phan, T.H., Yamamoto, K.: Resolving class imbalance in object detection with weighted cross entropy losses. arXiv preprint arXiv:2006.01413 (2020)
9. Simard, P.Y., Steinkraus, D., Platt, J.C.: Best practices for convolutional neural networks applied to visual document analysis. In: Icdar, vol. 2003-January, pp. 958–963. IEEE Computer Society (2003). DOI 10.1109/ICDAR.2003.1227801
10. Yang, J., Sharp, G., Veeraraghavan, H., van Elmpt, W., Dekker, A., Lustberg, T., Gooding, M.: Data from Lung CT Segmentation Challenge. The Cancer Imaging Archive. (2017). DOI http://doi.org/10.7937/K9/TCIA.2017.3r3fvz08
